# Visualization Environment for Federated Knowledge Graphs: Development of an Interactive Biomedical Query Language and Web Application Interface

**DOI:** 10.2196/17964

**Published:** 2020-11-23

**Authors:** Steven Cox, Stanley C Ahalt, James Balhoff, Chris Bizon, Karamarie Fecho, Yaphet Kebede, Kenneth Morton, Alexander Tropsha, Patrick Wang, Hao Xu

**Affiliations:** 1 Renaissance Computing Institute University of North Carolina at Chapel Hill Chapel Hill, NC United States; 2 CoVar Applied Technologies Durham, NC United States; 3 UNC Eshelman School of Pharmacy University of North Carolina at Chapel Hill Chapel Hill, NC United States

**Keywords:** knowledge graphs, clinical data, biomedical data, federation, ontologies, semantic harmonization, visualization, application programming interface, translational science, clinical practice

## Abstract

**Background:**

Efforts are underway to semantically integrate large biomedical knowledge graphs using common upper-level ontologies to federate graph-oriented application programming interfaces (APIs) to the data. However, federation poses several challenges, including query routing to appropriate knowledge sources, generation and evaluation of answer subsets, semantic merger of those answer subsets, and visualization and exploration of results.

**Objective:**

We aimed to develop an interactive environment for query, visualization, and deep exploration of federated knowledge graphs.

**Methods:**

We developed a biomedical query language and web application interphase—termed as Translator Query Language (TranQL)—to query semantically federated knowledge graphs and explore query results. TranQL uses the Biolink data model as an upper-level biomedical ontology and an API standard that has been adopted by the Biomedical Data Translator Consortium to specify a protocol for expressing a query as a graph of Biolink data elements compiled from statements in the TranQL query language. Queries are mapped to federated knowledge sources, and answers are merged into a knowledge graph, with mappings between the knowledge graph and specific elements of the query. The TranQL interactive web application includes a user interface to support user exploration of the federated knowledge graph.

**Results:**

We developed 2 real-world use cases to validate TranQL and address biomedical questions of relevance to translational science. The use cases posed questions that traversed 2 federated Translator API endpoints: Integrated Clinical and Environmental Exposures Service (ICEES) and Reasoning Over Biomedical Objects linked in Knowledge Oriented Pathways (ROBOKOP). ICEES provides open access to observational clinical and environmental data, and ROBOKOP provides access to linked biomedical entities, such as “gene,” “chemical substance,” and “disease,” that are derived largely from curated public data sources. We successfully posed queries to TranQL that traversed these endpoints and retrieved answers that we visualized and evaluated.

**Conclusions:**

TranQL can be used to ask questions of relevance to translational science, rapidly obtain answers that require assertions from a federation of knowledge sources, and provide valuable insights for translational research and clinical practice.

## Introduction

The semantic web is comprised of a collection of large graph databases of knowledge assertions enumerated as subject-predicate-object “triples” [1]. The subject and the object are considered unique entities that have globally unique identifiers. The predicate provides a relationship between the subject and the object. These assertions typically are derived from costly, manual digital curation of data sources in order to record both the assertion and the provenance of each assertion, although tools such as DataStaR have been developed to support efficiency and scale [2].

Recent work aims to semantically federate these “knowledge graphs” (KGs) in a manner that supports a unified query interface to address the heterogeneity of the corpus of knowledge sources [3]. These efforts have employed varied technological tools, including open web application programming interfaces (APIs), graph databases, and interface standards. The federated design of core semantic web technologies (such as SPARQL) has long acknowledged that the domain of all interconnected knowledge is too large to manage via a single monolithic database infrastructure with a single curatorial staff. Rather, the ability to allow multiple teams to work independently, while connecting them through semantic consensus, supports scale and the independence to experiment with new kinds of data not envisioned by a monolithic system. Federation provides such an environment to support a diverse community of collaborators, foster modular disciplinary specialization, and integrate multiple knowledge sources, both public and private.

However, federation presents challenges, in that any query system must provide semantic query planning to route queries to the appropriate knowledge sources, generate and evaluate answer subsets, and then merge the answer subsets into a semantically valid, coherent whole.

The Biomedical Data Translator program (hereinafter referred to as “Translator”) [4-6], funded by the National Center for Advancing Translational Sciences, National Institutes of Health, was created to address the challenge of query and interrogation across diverse data types and the many open data sets that are available but are not semantically compatible. The ability to interrogate relationships across the full spectrum of data types is critical in order to find answers to pressing translational questions. The Translator semantic informatics platform provides a comprehensive, unified semantic framework and approach to support such interrogation across disparate knowledge sources.

Herein, we present the Translator Query Language (TranQL) as a graph-oriented biomedical query language and web application to support query and visualization of semantically federated biomedical knowledge sources for deep, iterative exploration of query answers. TranQL leverages the semantic framework developed as part of the Translator program to support query across the Translator ecosystem.

## Methods

### Overview of TranQL Design

TranQL was designed to overcome the challenges in federating queries across heterogeneous distributed knowledge sources and merging answer subsets into a semantically cohesive whole. A key design feature is the adoption of a shared schema or namespace for navigating a globally agreed-upon conceptual structure that expresses entity types and the relationships between them. Such a schema accommodates queries that target subsets of the larger federated space and provides flexibility to extend domains or specialize within a specific domain. TranQL leverages the Biolink data model and the Translator Knowledge Graph Standard (KGS) API to implement this design feature. The TranQL query language and the TranQL web application are used for query execution and exploration of query results. These components are described in detail in the following sections.

### Biolink Data Model

The Biolink data model [7] provides the highest level of abstraction of biomedical concepts, thereby omitting the level of specificity elaborated by ontologies specific to biomedical domains such as those focused solely on “disease,” “phenotype,” or “anatomy.” Biolink is hierarchical and addresses both entities and relationships between them. TranQL uses the Biolink data model as an upper-level biomedical ontology to express concepts and relationships in the body of a knowledge query.

### Translator KGS API

The Translator KGS API [8] was developed as part of the Translator program and specifies a standard protocol for expressing a query as a graph of Biolink data elements that can be resolved into a KG consisting of nodes and edges from multiple knowledge sources and mappings between the KG and specific elements of the query. The Translator KGS API, therefore, enables a semantically coherent and technologically uniform query pattern over a federated, but otherwise heterogeneous, knowledge network.

### TranQL Query Language

**Figure 1:**
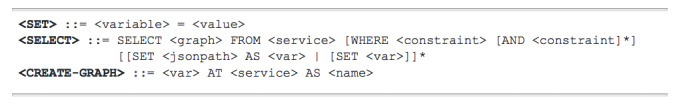
Structure of the query language.

The TranQL query language consists of a lexical analyzer, parser, and abstract syntax tree. The lexical analyzer recognizes a query language with the structure shown in Figure 1.

The “set” command assigns a value to a variable. The “select” command specifies a query graph of linked Biolink concepts. The query graph provides the framework for the resultant federated knowledge network. The “from” clause specifies the Translator KGS API. The invoked Translator KGS API endpoints return nodes and edges that conform to the semantic structure defined by the query graph. The optional “where” clause begins a list of constraints. Constraints can specify a value for an element of the query graph, supply options for the service invocation, or filter results. The “select” command may include a “set” command. For example, one form uses a JSON path query to extract an element of the resultant KG and assign it to a variable; another form assigns the entire KG to a variable. Finally, the “create graph” statement sends the resultant named KG to a service for storage.

### TranQL Web Application

The TranQL web application includes several components that together support interactive exploration: the TranQL query schema, TranQL backplane, and TranQL user interface (UI). The TranQL UI is a single-page web application that includes a query editor, cache function, graph visualization environment, answer viewer, and visualization controls.

#### TranQL Schema

The TranQL schema declares Translator KGS API endpoints and associates each one with a graph of the kinds of entity transitions it supports. These semantic transition “maps” are obtained from each endpoint. They describe the capabilities of endpoints in terms of the Biolink data model. Each endpoint is represented as a hierarchy in which the first level is an entity, the second is an entity, and the third is a relationship between the 2 entities. TranQL’s schema merges these transition maps into a unified KG. “Select” statements, containing the schema endpoint in the “from” clause, enable query planning. Planning compares each step in the query graph with the schema’s possible transitions to construct a plan for service invocations. The engine executes the plan by sending fragments of the query graph to those services able to complete the request. It then passes the results from one query segment to the next query segment and merges the composite results. Importantly, metadata indicating the provenance of each node and edge in the graph are preserved.

#### TranQL Backplane

The TranQL backplane is an OpenAPI implemented as a protocol normalization layer over the federated Translator KGS API endpoints. The backplane standardizes incompatibilities and separates the details of service invocations from the query language. When invoking backplane services, the “from” clause specifies an abbreviated syntax, thereby avoiding the need to specify the HTTP protocol, domain name, and port syntax to a specific service. The TranQL OpenAPI processes TranQL queries by parsing the query, planning service-specific questions that are implied by the query, executing those queries by invoking the backplane, collecting answers from services, and merging those answers into a single Translator KGS API–compliant response.

#### Query Editor and Cache Function

The query editor provides syntax highlighting, line numbers, and keyword autocompletion for the TranQL query language and the Biolink data model. The editor is implemented using CodeMirror [9], which provides the basis for many TranQL capabilities. Running a query retrieves a cached answer from a previous execution of the query or invokes one or more Translator KGS API endpoints. The resultant KG is rendered in a force-directed graph layout. Results are cached using the text of the query as a key. Changes to the query circumvent the cache. The query cache can be temporarily disabled or completely cleared via the settings dialog.

#### Graph Visualization Environment

The graph visualization environment uses the graphical processing unit (GPU)–accelerated 3D-rendering components based on WebGL [10] and Three.js [11]. These tools support fluid exploration of KGs comprised of tens of thousands of nodes. TranQL uses a configurable force-directed layout to control the distance between nodes, thereby providing a more intuitive visual organization of the KG. Nodes and edges are colored according to semantic type, as defined by the Biolink data model. Mouse events trigger the display of available information on each component of the KG. Deeper investigation of each node is supported by the “Select” mode. In “Navigation” mode, the selection of any node in the KG will center the selected node and orient the camera to look directly at it, thus obviating the need for incremental manual navigation.

#### Answer Viewer

The TranQL application integrates the ROBOKOP (Reasoning Over Biomedical Objects linked in Knowledge Oriented Pathways) answer viewer [12] to render a tabular view of the KG, as opposed to an interactive graph view. Both TranQL and ROBOKOP adhere to the Translator KGS API protocol, thereby allowing TranQL to send query results directly to the ROBOKOP answer viewer.

#### Visualization Controls

The “Settings” dialog provides 3 control panels for visualizations. The first panel allows users to select any of the 2-dimensional (2D), 3D, or virtual reality as the display style for the KG, with toggling to choose whether nodes and edges are colored by type. This panel also has controls for temporarily disabling the cache or clearing the cache entirely. A second panel provides 2 controls that relate to the structure of the KG. The first control configures a range of edge weights such that only edges with weights falling within the range will be rendered in the visualization. The second control configures a range of node connectivity such that only nodes with connections falling within the range will be rendered in the visualization. The third panel presents a list of checkboxes that correspond to knowledge sources that provide edges in the answer KG. The checkboxes allow users to select the preferred knowledge sources for rendering in the visualization.

## Results

### Overview of Use Case Results

We developed use cases in which a single query was posed to the TranQL UI and spanned federated knowledge managed by 2 independent Translator KGS API endpoints. The first endpoint was ICEES (Integrated Clinical and Environmental Exposures Service [13,14]. ICEES provides open access to clinical data derived from UNC Health that have been integrated with public data on environmental exposures. The second endpoint was ROBOKOP [12,15,16]. ROBOKOP is an open question–answering system that provides access to linked biomedical entities, such as “gene,” “chemical substance,” and “disease,” that are derived largely from curated public data sources.

### Use Case 1

**Figure 2:**
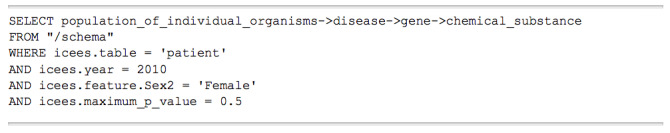
TranQL query for use case 1.

The first query asked in natural language, “What diseases are differentially associated with males and females, and what genes and chemical substances are associated with those diseases?” The overall intent of the query was to (1) validate TranQL by replicating established findings on sex differences and (2) discover chemicals or drugs that may be used to treat diseases that differentially affect males versus females. The natural language question is manually translated into the TranQL query language, and the resultant query is shown in Figure 2.

The first part of the query derives validation from ICEES on diseases differentially diagnosed in males versus females (ie, greater than chance); the second part of the question derives exploratory information from ROBOKOP on genes and chemical substances associated with those diseases. The “from” clause directs TranQL to conduct schema planning. The “icees“ prefixed parameters in the “where” clause are feature variables within ICEES and become options in the Translator KGS API protocol that are transmitted to the schema element designated by each parameter.

**Figure 3:**
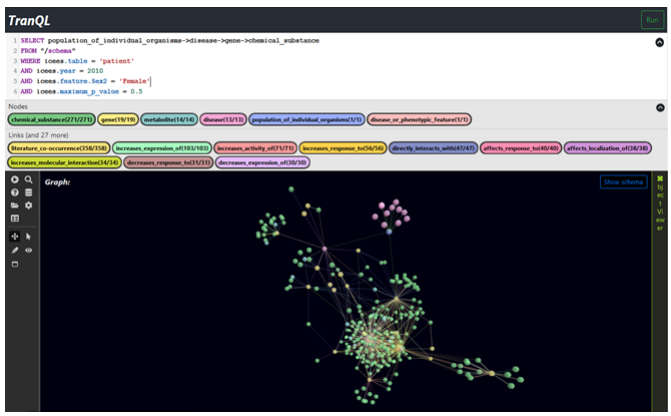
Use Case 1: example TranQL query and answer KG. The TranQL query was manually translated into the TranQL query language from a natural language query that asked, “What diseases are differentially associated with males and females, and what genes and chemical substances are associated with those diseases?”.

The TranQL query, as posed to the UI, and the resultant answer KG are shown in Figure 3. Note that nodes are color-coded according to entity type; links are similarly color-coded. Users can explore the answer KG using a variety of tools to enable zooming, rotation, choice of KG views (2D, 3D, and virtual reality), a tabular view of connected nodes, and a variety of other features.

**Figure 4:**
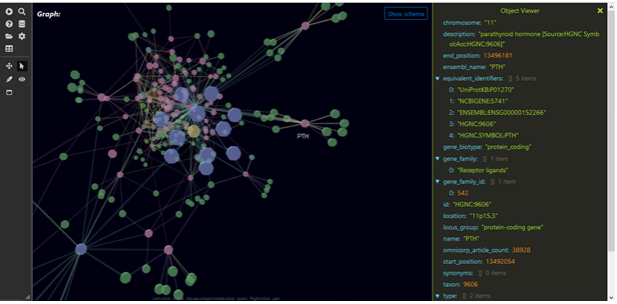
Metadata associated with the gene *PTH* (parathyroid hormone), which are found in the answer KG for the TranQL query shown in Figure 3.

One pathway in the answer KG shows that males and females are differentially diagnosed with ovarian cancer, a known female disease, thus validating the ability to extract factual information from ICEES using TranQL. The pathway traverses from ICEES to ROBOKOP to demonstrate an association between ovarian cancer and the gene *PTH* (parathyroid hormone), which ROBOKOP associates with the chemical substances—calcitriol, calcium atom, vitamin D, calcium carbonate, phosphane, adenine, phosphate, phosphorous, maxacalcitol, calciol, calcium, lithium hydride, and cinacalcet. Figure 4 highlights *PTH* and demonstrates the “object viewer” feature that allows users to review the metadata associated with the gene.

A quick Google search identifies several case reports describing hypercalcemia associated with parathyroid hormone in women with ovarian cancer, including a high-profile study by Nussbaum and colleagues [17] and a more recent one by Ma and colleagues [18]. These findings provide validation of TranQL and further suggest avenues for exploratory drug discovery in the treatment of ovarian cancer.

### Use Case 2

**Figure 5:**
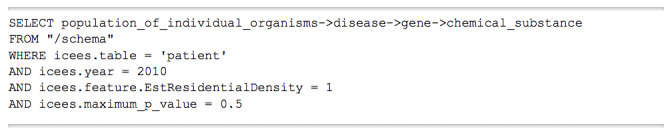
TranQL query for use case 2.

A second example query asked in natural language, “What diseases are differentially expressed in patients who live in rural versus urban regions, and what genes and chemical substances are associated with those diseases?” As with the first use case, the natural language query in this use case is manually translated into the TranQL query language, as shown in Figure 5.

**Figure 6:**
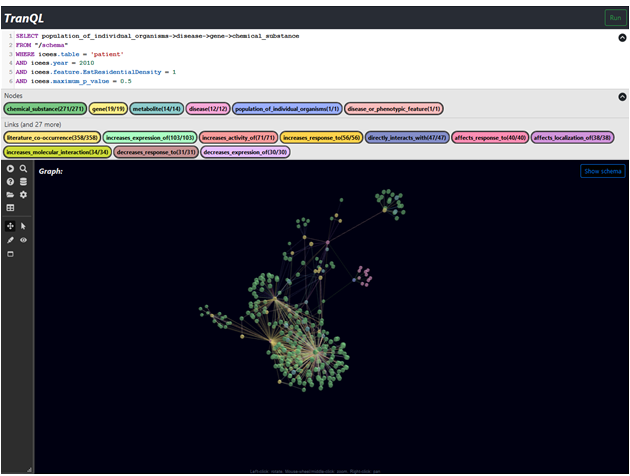
Use Case 2: example TranQL query and resultant KG. The TranQL query was manually translated into the TranQL query language from a natural language query that asked, “What diseases are differentially expressed in patients who live in rural versus urban regions, and what genes and chemical substances are associated with those diseases?”.

As with the first example query, this query also extracts clinical information on diseases from ICEES and biomedical information on genes and chemical substances from ROBOKOP. The query, as posed in the UI, and the resultant answer KG are shown in Figure 6.

**Figure 7:**
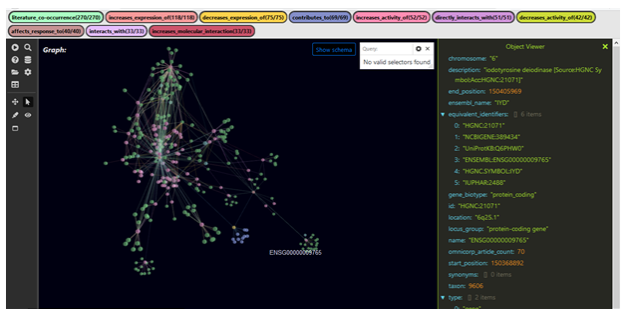
Metadata associated with the gene *IYD* (iodotyrosine deiodinase), which are found in the answer KG for the TranQL query shown in Figure 6.

One pathway through the resultant answer KG shows that croup is differentially diagnosed among patients who live in rural versus urban regions. The pathway traverses from ICEES to ROBOKOP and identifies the gene *IYD* (iodotyrosine deiodinase), as shown in Figure 7. ROBKOP associates *IYD* with the chemicals—polybrominated biphenyls, pentabromodiphenyl ether, halogenated diphenyl ethers, 2,2’,4,5’-tetrabromodiphenyl ether, 3,5-diiodo-L-tyrosine, triclosan, trihydroiodine, erythrosine, rose bengal, hydrogen iodide, benzbromarone, chlorobiphenyl, fenson, NADPH, NADP(+), flavin mononucleotide, and eosin B.

Several quick Google searches find that the majority of the identified chemicals represent hazardous substances (eg, polybrominated biphenyls) or food additives (eg, flavin mononucleotide) that differentially affect infants and young children, either due to enhanced toxicity or increased probability of exposure. For instance, polybrominated biphenyls are classified by the US Environmental Protection Agency as hazardous substances that were once used as flame retardants and plastic additives [19]. Although they are now prohibited, the compounds remain in the environment and differentially impact infants and young children. Similarly, croup differentially affects infants and young children [20], thus providing a plausible explanation for the association and further suggesting that the identified compounds may also contribute directly to croup.

## Discussion

### Principal Findings

We have created a biomedical query language—TranQL—to support query, visualization, and exploration of federated biomedical KGs. TranQL leverages the semantic framework and approach developed as part of the Translator program to semantically integrate large biomedical KGs, thereby allowing users to pose challenging translational queries that span multiple knowledge sources, rapidly explore the results, and derive valuable insights for translational research. Importantly, we have validated TranQL using 2 driving use cases and queries that span clinical knowledge sources and observational biomedical knowledge sources.

The Translator semantic platform allows users to interrogate relationships across the full spectrum of data types, without needing to manually search through individual databases and data sets that exhibit varying levels of semantic inference rules and linkages among entities. Moreover, the Translator framework supports consistent data linkage and semantic resolution across data sources due to adoption of the Biolink data model and the Translator KGS API specification as well as mappings to relevant biomedical ontologies, such as Monarch Disease Ontology and Human Phenotype Ontology.

In addition to leveraging the Translator framework and approach, TranQL provides several other capabilities. First, TranQL offers a domain-specific query language that makes iterative query and result exploration practical and approachable by users without any experience of software development. TranQL also provides a layer of abstraction for accessing a federation of services, and the interactive web interface offers graphical and tabular visualizations of query answers. Moreover, although not demonstrated herein, TranQL is designed to scale to federations involving more than 2 knowledge sources.

### Limitations

TranQL has several limitations that should be considered. First, TranQL is constrained in expressivity, in that the tool can only represent linear paths through KGs, as opposed to more complex structures. Second, TranQL is limited in the number of available features and the ability to perform operations. Third, users are required to manually map natural language queries to the TranQL query language. Automated machine translation of natural language queries to machine queries remains a major challenge within the Translator program and elsewhere, primarily due to ambiguity related to intent and context [21]. Fourth, unstructured data likewise remain a major challenge within the Translator program and elsewhere, although progress has been made in certain areas. For instance, Translator team members are developing tools to handle notoriously challenging types of unstructured data such as clinical laboratory measures, including the LOINC2HPO tool that maps clinical laboratory measures to the human phenotype ontology [22]. Fifth, TranQL answer sets can be challenging to navigate, especially when a large number of nodes and edges are returned to users. This remains a challenge with KGs in general, although we are considering approaches to provide additional views such as the rudimentary tabular view that is under development. Finally, although KGs are a common approach to knowledge representation, they are by no means the only approach. For instance, knowledge fusion patterns are gaining in popularity as a new form of knowledge representation [23].

Despite these limitations, we believe that TranQL will find broad adoption due to its ability to support speed to discovery of insights to complex translational questions and generate mechanistic hypotheses for subsequent investigation and testing.

### Future Directions

TranQL is under active development, with performance and feature enhancements deployed regularly. Planned feature enhancements include a more user-friendly interface and additional visualization capabilities to support answer exploration. We are working with subject matter experts to iteratively improve the TranQL UI and other features of the interactive web application. We are also working to improve the robustness of query answers. For instance, we are developing approaches to harmonize across entity identifiers in order to accommodate the disparate identifier systems adopted by different Translator KGS API endpoints and enable more complete federation of those knowledge sources.

### Availability

TranQL is publicly available on its website [24]. Software code and instructions are available under the MIT open software license at a GitHub repository [25]. We encourage users to post issues to the TranQL GitHub repository and report identified software bugs or request new features and capabilities. Additional information and example queries can be found on the webpages of TranQL API [26] and the Translator program [27].

## Abbreviations

2D: 2-dimensional
API: application programming interface
GPU: graphical processing unit
ICEES: Integrated Clinical and Environmental Exposures Service
IYD: iodotyrosine deiodinase
KG: knowledge graph
KGS: knowledge graph standard
PTH: parathyroid hormone
ROBOKOP: Reasoning Over Biomedical Objects linked in Knowledge Oriented Pathways
TranQL: Translator Query Language
UI: user interface
